# Electrical storm in a patient with Charcot–Marie–Tooth disease treated with radiofrequency ablation and subsequent serious complication of implantable cardioverter defibrillator implantation: a case report

**DOI:** 10.1093/ehjcr/ytae673

**Published:** 2024-12-18

**Authors:** Jiří Vrtal, Jiří Plášek, Jan Václavík, David Šipula, Jozef Dodulík

**Affiliations:** Department of Internal Medicine and Cardiology, Faculty of Medicine, University Hospital Ostrava, University of Ostrava, 17. listopadu 1790, 708 52 Ostrava, Czech Republic; Research Center for Internal and Cardiovascular Diseases, Faculty of Medicine, University of Ostrava, 17. listopadu 1790, 708 52 Ostrava,Czech Republic; Department of Internal Medicine and Cardiology, Faculty of Medicine, University Hospital Ostrava, University of Ostrava, 17. listopadu 1790, 708 52 Ostrava, Czech Republic; Research Center for Internal and Cardiovascular Diseases, Faculty of Medicine, University of Ostrava, 17. listopadu 1790, 708 52 Ostrava,Czech Republic; Department of Internal Medicine and Cardiology, Faculty of Medicine, University Hospital Ostrava, University of Ostrava, 17. listopadu 1790, 708 52 Ostrava, Czech Republic; Research Center for Internal and Cardiovascular Diseases, Faculty of Medicine, University of Ostrava, 17. listopadu 1790, 708 52 Ostrava,Czech Republic; Department of Internal Medicine and Cardiology, Faculty of Medicine, University Hospital Ostrava, University of Ostrava, 17. listopadu 1790, 708 52 Ostrava, Czech Republic; Department of Internal Medicine and Cardiology, Faculty of Medicine, University Hospital Ostrava, University of Ostrava, 17. listopadu 1790, 708 52 Ostrava, Czech Republic

**Keywords:** Charcot–Marie–Tooth, Ventricular tachycardia, Case report, Electric storm, ICD

## Abstract

**Background:**

Charcot–Marie–Tooth is the most common inherited neuromuscular disorder. Rarely, it can be associated with heart failure and various arrhythmic disturbances. This case illustrates the challenges of making decisions to prevent sudden cardiac death in a patient with Charcot–Marie–Tooth disease.

**Case summary:**

A 69-year-old male with a history of Type 1A Charcot–Marie–Tooth disease was admitted due to repetitive runs of ventricular tachycardia. Twelve-lead electrocardiogram, echocardiography, selective coronary angiography, and cardiac magnetic resonance did not clarify the cause of the electrical storm. As conservative therapy was not successful, radiofrequency ablation was chosen to treat the electrical storm. After this procedure, implantable cardioverter defibrillator (ICD) was implanted. The follow-up revealed severe perforation by the ventricular lead. An extraction was performed with no complications and a new lead was immediately implanted. The patient remains asymptomatic. Three episodes of non-sustained ventricular tachycardia were recorded during the last follow-up.

**Discussion:**

This case illustrates the challenges of making decisions to prevent sudden cardiac death in a patient with Charcot–Marie–Tooth disease after successful ablation for electrical storm. Due to a lack of evidence, atypical origin of arrhythmia, and clinical presentation, we did not consider this as idiopathic arrhythmia and decided to implant an ICD, which was complicated by severe perforation by the lead. Specific recommendations for preventing sudden cardiac death in rare cardiac conditions, such as Charcot–Marie–Tooth disease, still need to be refined.

**Learning points**

- To understand the relation between Charcot–Marie–Tooth disease and arrhythmias.
- To highlight the dilemma of whether focal arrhythmia in Charcot–Marie–Tooth may be perceived as idiopathic.

## Introduction

Charcot–Marie–Tooth (CMT) is the most common inherited neuromuscular disorder.^1–3^ Charcot–Marie–Tooth disease can be associated with heart failure and various arrhythmic disturbances.^4^ However, the causal relationship between cardiac abnormalities and the mutation is still unclear. This case illustrates the challenges in making decisions to prevent sudden cardiac death (SCD) in a patient with CMT disease after successful ablation for electrical storm.^5^

## Summary figure

**Table:** 

Time	Event
Day 0	Admitted to the intensive care unit for repetitive runs of ventricular tachycardia with suspected origin in the posteromedial papillary muscle. Initial pharmacotherapy with little effect. Echocardiography with normal systolic function of both ventricles. Coronary angiography with no signs of obstructive coronary disease.
Day 1	Pharmacological sedation and left ganglion stellatum blockade to suppress runs of ventricular tachycardia. Cardiac magnetic resonance without signs of oedema, scar, or gadolinium enhancement.
Day 2	Radiofrequency ablation of electric storm with origin surprisingly in anterolateral papillary muscle due to its slightly abnormal position.
Day 6	Implantation of ICD in secondary prevention of sudden cardiac death.
Day 7	Patient discharged.
2 months	First follow-up with very low impedance, no capture, and no sensing of intrinsic ventricular activity during interrogation. Computed tomography revealed perforation of lead through the ventricle, pericardium, and intercostal muscle. Extraction procedure with immediately implanted new lead to different position.
4 months	Second follow-up with good parameters of lead. In the device memory, three episodes of non-sustained ventricular tachycardia with a maximum of 16 beats.

## Case presentation

**Figure 1:**
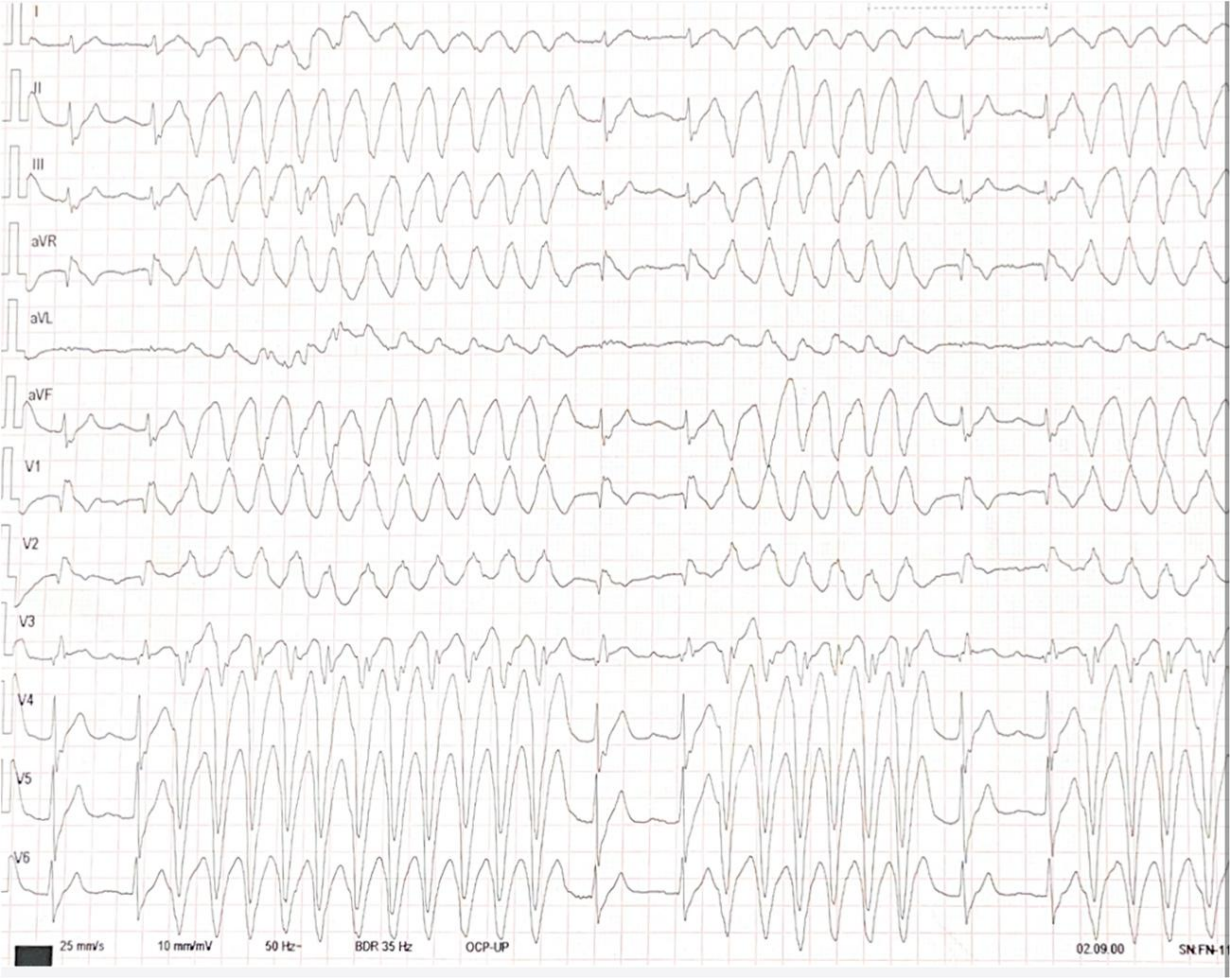
Initial electrocardiogram showing the run of ventricular tachycardia.

A 69-year-old male with a history of Type 1A CMT disease with a confirmed peripheral myelin protein 22 (PMP22) mutation, hepatopathy, and arterial hypertension with very good premorbid functional status was brought by ambulance to the emergency department for several hours lasting of dizziness, fatigue, and chest pain. His medication consisted of 2.5 mg bisoprolol once daily. During the clinical examination, the patient had pitting oedema of the ankles. The patient's ECG showed repetitive runs of ventricular tachycardia (VT), with QRS morphology suggesting an origin in the posteromedial papillary muscle (*Figure 1*). However, no signs of acute coronary syndrome, long-QT syndrome, or other conditions that could cause such runs were observed on the resting 12-lead ECG. The patient was admitted to the cardiac intensive care unit, where initial laboratory tests revealed moderately elevated levels of N-terminal pro-B-type natriuretic peptide (1208 ng/L) and slightly elevated highly sensitive troponin I (207 ng/L) with the absence of further dynamic of troponin level. Plasma levels of all sampled electrolytes including natrium, potassium, or magnesium were in the normal range. Chest X-ray did not detect any pathology. Bedside transthoracic echocardiography revealed normal ventricle systolic function and moderate mitral valve regurgitation. Finally, selective coronary angiography revealed no signs of obstructive coronary disease. For repeated episodes of haemodynamically tolerated VT, intravenous amiodarone and metoprolol were administered but showed little effect. To address the continued episodes of VT, the patient underwent left ganglion stellate blockade using 8 mL of 1% bupivacaine under direct ultrasound guidance. Mild sedation with midazolam was also initiated to suppress sympathomimetic activity. This therapy slightly reduced the burden of the VT episodes and allowed cardiac magnetic resonance to be performed. Cardiac magnetic resonance ruled out other possible causes of the VT, as there were no signs of oedema, scars, or late gadolinium enhancement present. As conservative therapy was not successful, radiofrequency ablation was chosen to treat the electrical storm. Whole procedure was guided by the intracardiac echocardiography, which is essential in papillary muscle VT ablation since papillary muscles are very mobile structures and both fluoroscopy and electroanatomic maps are mostly insufficient to guide the procedure. We used one steerable transseptal sheath (Agilis NxT™ large curl, Abbott, MN, USA) and irrigated-tip ablation catheter (TactiCath™, Abbott, MN, USA).

**Figure 2:**
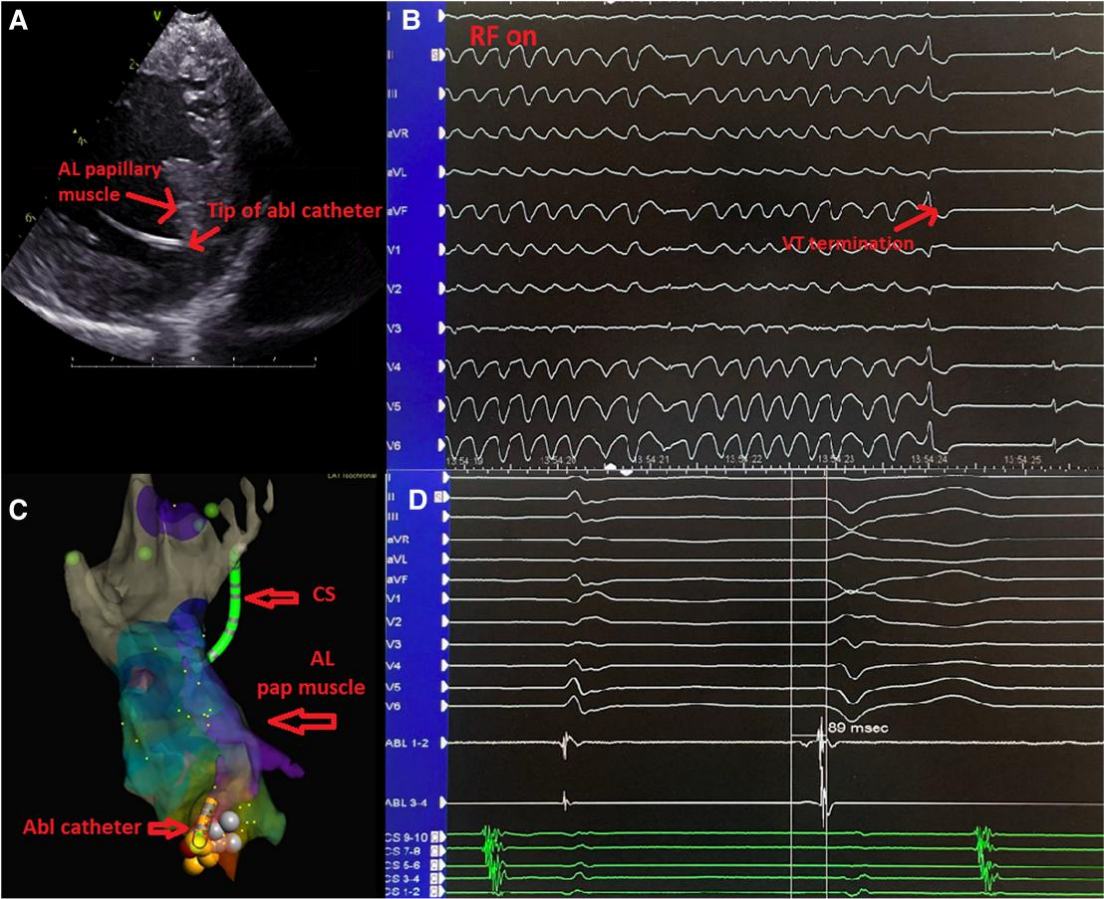
(*A*) Intracardiac echocardiography showing the ablation catheter’s position at the base of the anterolateral papillary muscle. (*B*) Termination of ventricular tachycardia during ablation. (*C*) Local activation time map demonstrating activation in the anterolateral papillary muscle. (*D*) Intracardial signals showing the earliest signals in the ablation catheter. Abl, ablation; AL, anterolateral; AP, anteroposterior; CS, coronary sinus.

A local activation map was created of premature ventricular complexes (PVCs) with the same morphology on 12-lead ECG as clinical runs of VT, and it showed the earliest activation at the posterior base of the anterolateral papillary muscle rather than the posteromedial papillary muscle (*Figure 2C*). Notably, also the pacemap from this spot was similar to the clinical VT.

The energy was titrated up to 40 W in the temperature-controlled mode with maximum temperature of 50°C. Radiofrequency energy was applied for a maximum of 60 s per target site depending also on the impedance drop and electrogram abatement. The difference between the ‘typical’ ECG pattern for papillary muscle PVC and the true origin of the VT can be explained by the relatively small left ventricle and a slightly abnormal position of the anterolateral papillary muscle, which was more posterior than usual on intracardiac echocardiography (*Figure 2A*). During the radiofrequency ablation procedure, spontaneously induced VT terminated, and no further ectopic activity was observed (*Figure 2B*). After the procedure, the patient did not experience any more PVCs or VT. Treatment with amiodarone was discontinued.

**Figure 3:**
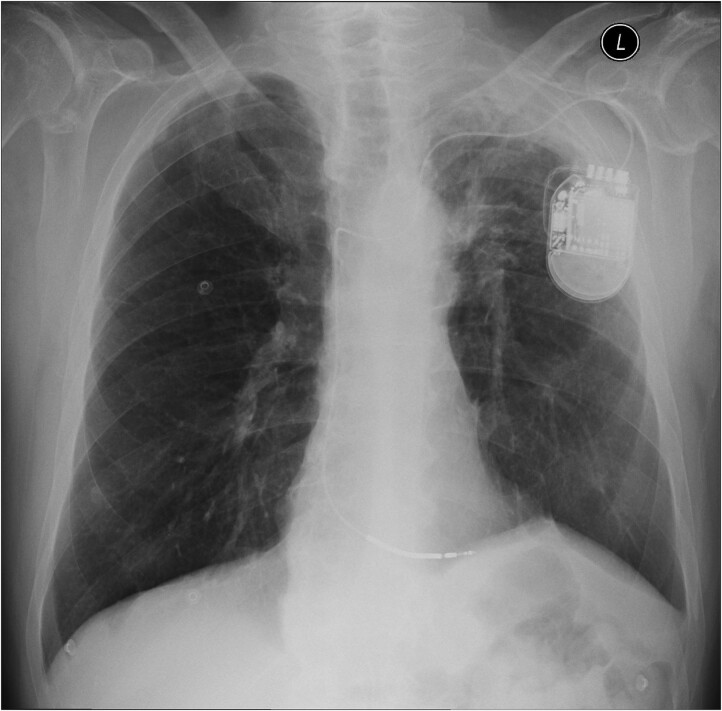
Chest X-ray (anteroposterior view) after the first implantation.

**Figure 4:**
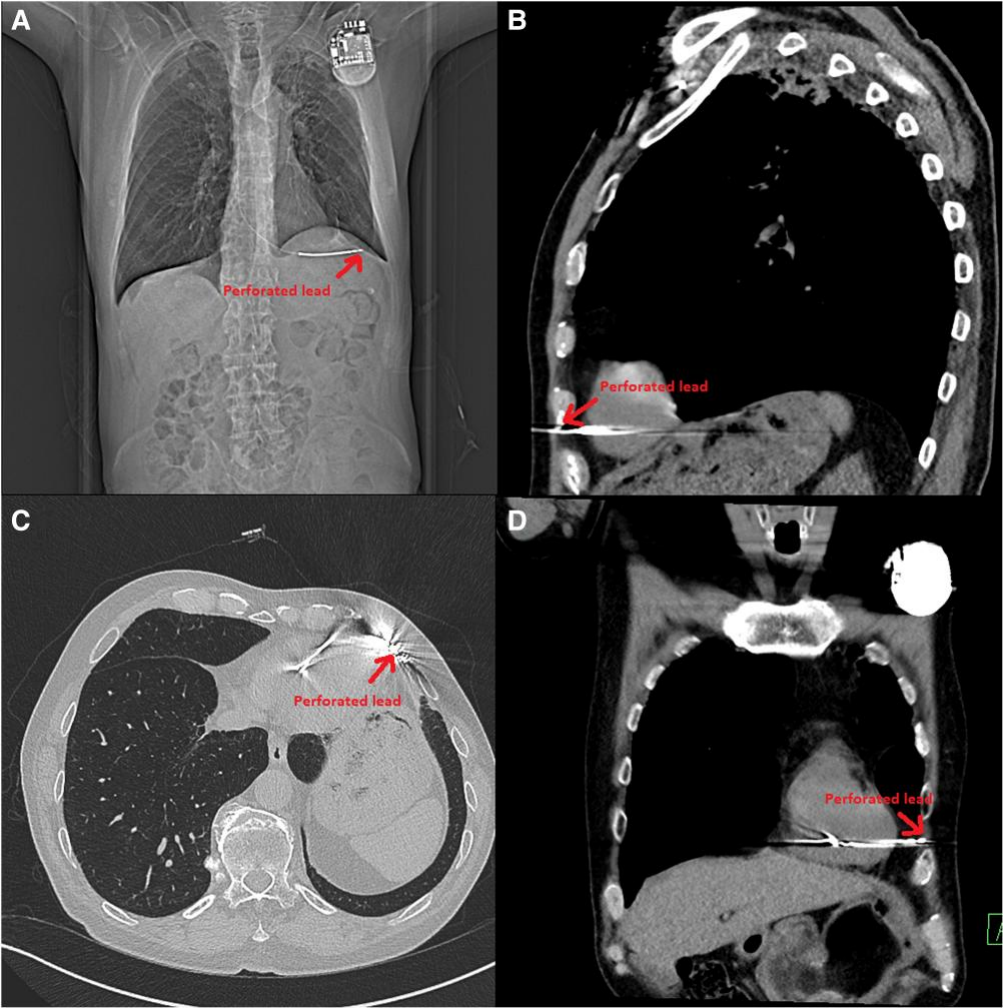
Computed tomography showing lead perforation through the pericardium to the intercostal muscle. (*A*) Initial topogram. (*B*) Sagittal view. (*C*) Transversal view. (*D*) Frontal view.

**Figure 5:**
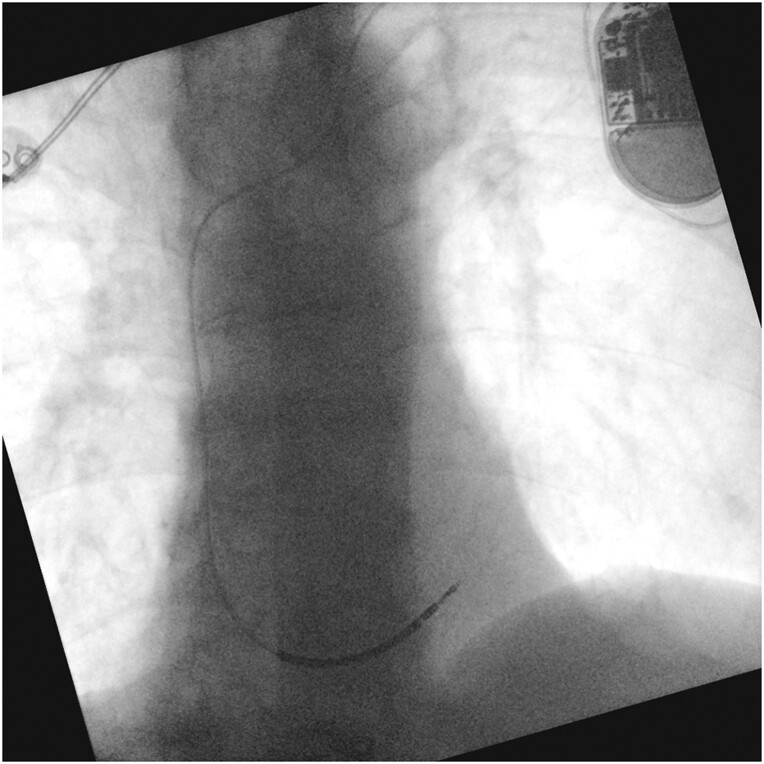
Periprocedural X-ray (anteroposterior view) during the second implantation.

After the successful radiofrequency ablation in the absence of structural heart disease, we hesitated whether or not to implant an implantable cardioverter defibrillator (ICD). After discussion, the factors in favour of implantation prevailed, so we decided for implantation. The procedure was performed using the standard transvenous approach, and the defibrillation lead was placed to where the basal septum of the right ventricle was thought to be located with excellent parameters (*Figure 3*). The patient was discharged the following day, and a regular follow-up was scheduled 6 weeks later. During this follow-up, the ICD interrogation revealed very low impedance, no effective stimulation of the right ventricle even with the highest possible voltage and stimulation width, and no sensing of intrinsic ventricular activity. Unexpectedly, no symptoms, such as chest pain or dyspnoea, were present. The patient’s blood pressure was also in the normal range, and the ECG showed sinus rhythm with a normal heart rate. An X-ray visualized possible lead perforation, whereas echocardiography showed no pericardial effusion. Computed tomography revealed that the lead passed through the right ventricle, pericardium, and intercostal muscles, causing a perforation (*Figure 4*). Extraction was performed the next day with the backup of a cardiac surgeon. There were no complications during the procedure and no haemopericardium after the procedure. The extraction procedure was followed by the immediate implantation of a new transvenous lead in a different position more towards the septum (*Figure 5*). During regular follow-up after reimplantation, three episodes of non-sustained VT were noted. The episodes were short, with a maximum duration of 5 s. The dose of the beta blocker was increased (metoprolol from 50 to 100 mg), and the electrolytes were checked, but there was no abnormality. Based only on the ICD-saved episode, we cannot confirm that these episodes were of the same origin as the initial arrhythmia. Otherwise, the ICD follow-up was unremarkable and the patient functional status was very good. To detect other mutations associated with a higher risk of SCD, genetic testing was performed again, confirming the known PMP22 mutation and ruling out other mutations.

## Discussion

Charcot–Marie–Tooth disease is the most common inherited neuromuscular disorder, with onset usually in the first two decades of life and subsequently slow progression over decades.^1,2^ Symptoms and signs indicative of CMT disease include pes cavus, difficulty with walking and hand manipulation, muscle cramps, and cold feet.^3^ Rarely, CMT disease can be associated with heart failure and various arrhythmic disturbances due to the PMP22 mutation in Type 1A CMT hereditary neuropathy.^4,6–8^ Less common Type 2 CMT disease can be associated with a mutation in the LMNA gene and can lead to arrhythmogenic right ventricular cardiomyopathy.^9,10^ Similarities in the pathological mechanisms of BAG3-induced dilated cardiomyopathy and Type 2 CMT have been described.^11^ However, the causal relationship between the cardiac abnormalities and mutation is still unclear. To understand the relation between CMT disease and arrhythmias, we highlight the dilemma of whether focal arrhythmia in CMT may be perceived as idiopathic. In general, arrhythmias are common and often the first manifestation of neuromuscular disorder. The present guidelines on VT management address primarily myotonic dystrophy, Duchenne/Becker and Emery–Dreifuss dystrophies.^12^ Even though there are serious differences in these dystrophies and CMT, we based our decision process on neuromuscular disorders as a group. We decided to implant an ICD because of the presence of neuromuscular disorder and the absence of specific guidelines for CMT. The origin of VT in our case was atypical. Most idiopathic VT originates in the right ventricular outflow tract, left ventricular outflow tract, or fascicles. Additionally, the clinical presentation with repetitive runs of VT leading to heart failure is not typical for idiopathic VT. We have also consulted the neurological team to share their experience on this particular disorder, and they confirmed the uncertainty of arrhythmic risk and supported our approach. Therefore, taking into account all the factors, we do not believe it was idiopathic VT and therefore we implanted an ICD.

Perforation by the defibrillation lead early after implantation is a rare but severe complication, making the decision about implantation even more challenging. In cases where there may be a higher risk of lead perforation such as low body mass index (BMI) or rotated heart, care should be taken when positioning the lead including the use of multiple X-ray views from different projections. After this event, it is questionable whether a new transvenous system should be implanted or a subcutaneous system should be used, or if the patient should be left without an ICD. In this case, it seems that implanting a subcutaneous ICD is the best option for a patient who does not require cardiac pacing. The main concern against utilizing a subcutaneous ICD was the patient's asthenic constitution with BMI of 20.6. Taking into account the patient’s preferences after thoroughly discussing various possibilities including subcutaneous ICD, we decided to implant a new transvenous system instead. In our country, the wearable cardioverter defibrillator is not available. In general, CMT has moderate to good prognosis based on specific mutation in terms of life expectancy.^3^ From the cardiological perspective, we markedly decreased potential arrhythmic risk since initially the electric storm could have easily lead to premature death. Nevertheless, specific recommendations for rare cardiac conditions, such as CMT, still need to be made in order to meet the specific needs of these patients.

## Data Availability

The data underlying this article will be shared on a reasonable request to the corresponding author.

## Lead author biography

Jiří Vrtal graduated at Palacky University in Olomouc in 2014 and completed his cardiology residency at University Hospital in Ostrava in 2019. He is interested in implanting procedures and also in electrophysiology.
